# Identification of Actionable Fusions as an Anti-EGFR Resistance Mechanism Using a Circulating Tumor DNA Assay

**DOI:** 10.1200/PO.19.00141

**Published:** 2019-10-03

**Authors:** Katherine Clifton, Thereasa A. Rich, Christine Parseghian, Victoria M. Raymond, Arvind Dasari, Allan Andresson Lima Pereira, Jason Willis, Jonathan M. Loree, Todd M. Bauer, Young Kwang Chae, Gary Sherrill, Paul Fanta, Axel Grothey, Andrew Hendifar, David Henry, Daruka Mahadevan, Mohammad Amin Nezami, Benjamin Tan, Zev A. Wainberg, Richard Lanman, Scott Kopetz, Van Morris

**Affiliations:** ^1^ The University of Texas MD Anderson Cancer Center, Houston, TX; ^2^ GuardantHealth, Redwood City, CA; ^3^ BC Cancer, Vancouver, British Columbia, Canada; ^4^ Tennessee Oncology Sarah Cannon Research Institute, Nashville, TN; ^5^ Northwestern University Feinberg School of Medicine, Chicago, IL; ^6^ Cone Health Cancer Center, Greensboro, NC; ^7^ University of San Diego Moores Cancer Center, La Jolla, CA; ^8^ The University of Tennessee West Cancer Center, Memphis, TN; ^9^ Cedars-Sinai Medical Center, Los Angeles, CA; ^10^ University of Pennsylvania, Philadelphia, PA; ^11^ The University of Arizona Cancer Center, Tucson, AZ; ^12^ Pacific Medical Center of Hope, Fresno, CA; ^13^ Washington University School of Medicine, St Louis, MO; ^14^ University of California Los Angeles, Los Angeles, CA

## Abstract

**PURPOSE:**

Gene fusions are established oncogenic drivers and emerging therapeutic targets in advanced colorectal cancer. This study aimed to detail the frequencies and clinicopathological features of gene fusions in colorectal cancer using a circulating tumor DNA assay.

**METHODS:**

Circulating tumor DNA samples in patients with advanced colorectal cancer were analyzed at 4,581 unique time points using a validated plasma-based multigene assay that includes assessment of fusions in *FGFR2*, *FGFR3*, *RET*, *ALK*, *NTRK1*, and *ROS1.* Associations between fusions and clinicopathological features were measured using Fisher’s exact test. Relative frequencies of genomic alterations were compared between fusion-present and fusion-absent cases using an unpaired *t* test.

**RESULTS:**

Forty-four unique fusions were identified in 40 (1.1%) of the 3,808 patients with circulating tumor DNA detected: *RET* (n = 6; 36% of all fusions detected), *FGFR3* (n = 2; 27%), *ALK* (n = 10, 23%), *NTRK1* (n = 3; 7%), *ROS1* (n = 2; 5%), and *FGFR2* (n = 1; 2%). Relative to nonfusion variants detected, fusions were more likely to be subclonal (odds ratio, 8.2; 95% CI, 2.94 to 23.00; *P* < .001). Mutations associated with a previously reported anti–epidermal growth factor receptor (anti-EGFR) therapy resistance signature (subclonal *RAS* and *EGFR* mutations) were found with fusions in *FGFR3* (10 of 12 patients), *RET* (nine of 16 patients), and *ALK* (seven of 10 patients). For the 27 patients with available clinical histories, 21 (78%) had EGFR monoclonal antibody treatment before fusion detection.

**CONCLUSION:**

Diverse and potentially actionable fusions can be detected using a circulating tumor DNA assay in patients with advanced colorectal cancer. Distribution of coexisting subclonal mutations in *EGFR*, *KRAS*, and *NRAS* in a subset of the patients with fusion-present colorectal cancer suggests that these fusions may arise as a novel mechanism of resistance to anti-EGFR therapies in patients with metastatic colorectal cancer.

## INTRODUCTION

Fusions resulting in activation of proto-oncogenes lead to pathologic proliferation in a variety of malignancies and can serve as potential therapeutic targets.^1,2^ Although selective kinase inhibitors have become standard-of-care therapies for *ALK-* and *ROS1-*rearranged non–small-cell lung cancers (NSCLCs), no US Food and Drug Administration–approved targeted therapies for fusions in colorectal cancer (CRC) were available until the recent approval of larotrectinib for any advanced solid tumor with *NTRK* fusions.^3^ In two small series, the ALK inhibitors ceritinib and entrectinib demonstrated benefit in patients with CRC harboring *ALK* fusions.^4,5^ In addition, rearranged during transfection (RET) inhibitors have shown preclinical promise in *RET* fusions both in vitro and in vivo for RET-fusion CRC.^6,7^ Using tissue-based assays, fusions have been reported in approximately 1% of patients with CRC but are more common in right-sided, *RAS* wild-type, microsatellite instability–high (MSI-H) colon cancers.^8-12^ However, no studies to date have comprehensively described the prevalence and genomic landscape of fusions in CRC using circulating tumor DNA (ctDNA).

**CONTEXT**

**Key Objective**
Using a circulating tumor DNA assay, we aimed to describe the frequencies and clinicopathological features of gene fusions in patients with advanced colorectal cancer.
**Knowledge Generated**
A circulating tumor assay was able to detect actionable fusions in patients with colorectal cancer at a prevalence of 1.1%. Genomic signatures previously associated resistance to anti–epidermal growth factor receptor therapies were found in patients with fusions.
**Relevance**
Coexisting subclonal mutations in patients with fusion-present colorectal cancer implicate fusions as a previously unreported, novel mechanism of resistance to anti–epidermal growth factor receptor therapies in patients with metastatic colorectal cancer.

When measured by ctDNA, early truncal mutations tend to occur at higher variant allele fractions (VAFs) compared with mutations acquired later in disease progression.^13^ ctDNA may thereby uncover the genomic evolution of mechanisms of treatment resistance, because subclonal mutations not initially detected in primary tumor specimens may become detectable after selective pressure of targeted therapies.^14^ For example, using ctDNA assays, *KRAS*, *NRAS*, *MET*, *ERBB2*, *EGFR*, *FGFR1*, and *MAP2K1* mutations have been identified as mechanisms of resistance to anti-EGFR antibody therapy in patients with CRC.^15-20^ Activating fusions have been found to be associated with resistance to EGFR-targeted therapies in several malignancies, including NSCLC and head and neck cancer.^21-24^

To our knowledge, no prior studies have detailed the use of a ctDNA assay in a large series for detection of oncogenic fusions in CRC. Here, we aimed to use next-generation sequencing (NGS) data from a ctDNA assay to expand the clinical use of fusion testing in a cohort of patients with advanced and typically pretreated CRC.

## METHODS

### Patient Population

A cohort of 4,289 consecutive patients with stage III or IV CRC underwent molecular profiling at 4,581 unique time points between February 2015 and December 2017 using a validated plasma-based 68-, 70-, or 73-gene ctDNA NGS assay (Guardant360, Guardant Health, Redwood City, CA), as previously detailed.^25,26^ This assay was performed in a Clinical Laboratory Improvement Amendments–certified, College of American Pathologists–accredited, and New York State Department of Health–approved setting using a targeted digital sequencing panel with the ability to detect single-nucleotide polymorphisms, insertions/deletions (indels), amplifications, and fusions. The 68-gene panel included *ALK*, *RET*, *ROS1*, and *NTRK1* fusions, and the 70- and 73-gene panels also tested for *FGFR2* and *FGFR3* fusions (Appendix Table A1). There was no difference in the exon coverage of *KRAS*, *NRAS*, *PIK3CA*, *BRAF*, or *EGFR* among these three panels. Germline variants were filtered out as previously described.^27^ The reportable range for single nucleotide variants (SNVs), indels, fusions, and amplifications is greater than 0.04% per two molecules, greater than 0.02% per one molecule, greater than 0.04% per two molecules, and greater than 2.12 copies, respectively, with a greater than 99.9999% per-position analytic specificity.^26^ Clinical information was obtained from test request forms and confirmed by pathology and medical reports and from treating clinicians when available. This research was approved by the Quorum Institutional Review Board for the generation of de-identified data sets for research. All work was conducted in accordance with the Declaration of Helsinki. Human investigations were performed after approval by a local human investigations committee and in accordance with an assurance filed with and approved by the Department of Health and Human Services, where appropriate.

### ctDNA Assay Analysis

VAF was calculated as the ratio of the number of ctDNA molecules harboring a mutation relative to the total number of molecules (variant plus wild type) for a given gene locus. To annotate a given alteration by clonality, relative VAF (rVAF) was assessed by normalizing the VAF to the maximum VAF of all aberrations detected within a given plasma sample, adjusting for copy number amplification as previously described.^13^ For the purpose of this study, clonal aberrations were defined as rVAF of 0.5 to 1, subclonal aberrations as rVAF between 0.1 and 0.5, and subclonal minor as rVAF less than 0.1.^13^

Associations between the presence of fusions and clinicopathological features were evaluated using a Fisher’s exact test (SPSS, version 24.0; La Jolla, CA). Relative frequencies of genomic alterations (point mutations, indels, and splice variants) were compared between fusion-present and fusion-absent cases using an unpaired *t* test.

## RESULTS

### Occurrence of Fusions in a ctDNA Assay

**FIG 1.**
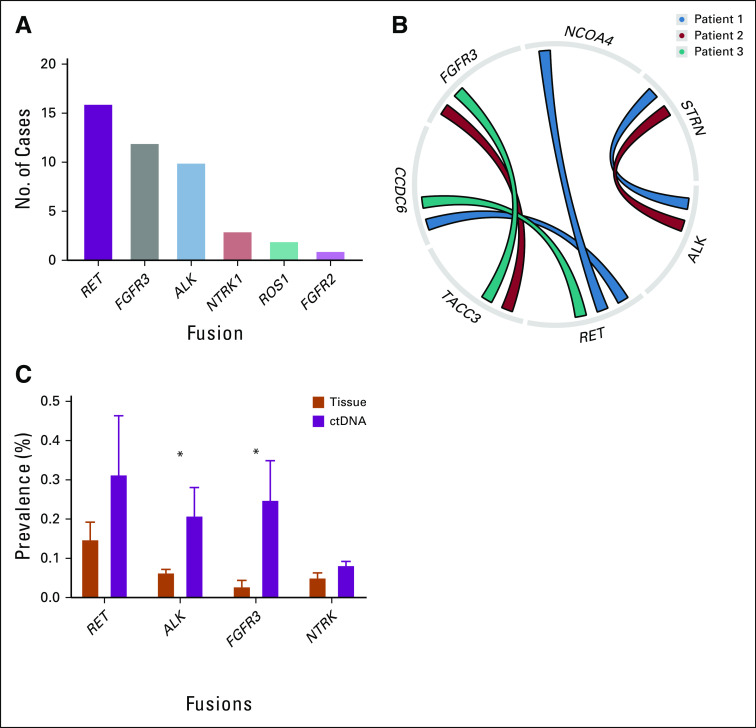
Prevalence of fusions with circulating tumor DNA (ctDNA). (A) Overall prevalence of fusions. (B) Specimens with co-occurring fusions. (C) Fusion prevalence in ctDNA-based assay compared with tissue-based assay with 95% upper CIs. (*) Indicates fusions with statistically significant differences in prevalence between tissue and ctDNA.

The median age at time of ctDNA testing was 59 years (interquartile range, 50-69 years). A total of 1,909 patients (44.5%) were female. Of the 3,808 patients with detectable alterations at any time point (Fig 1A), 44 unique fusions were reported in 40 patients (1.1% prevalence). These fusions detected *RET* (n = 16; 36% of all fusions detected), *FGFR3* (n = 12; 27%), *ALK* (n = 10; 23%), *NTRK1* (n = 3; 7%), *ROS1* (n = 2; 5%), and *FGFR2* (n = 1; 2%). When examining the prevalence of fusions by rearrangement partner, the most commonly detected fusions were the *FGFR3-TACC3* (n = 12) and *NCOA4-RET* (n = 9) fusions (Appendix Table A2). Co-occurring fusions were found in three of 40 patients (Fig 1B).

The prevalences of *ALK* and *FGFR3* fusions were significantly higher in this ctDNA cohort compared with a previously reported cohort of 4,422 CRC tissue specimens undergoing comprehensive NGS genomic profiling (*P* = .04, *P* = .01, respectively).There was no difference in frequencies of *RET* or *NTRK* fusions between ctDNA and tissue assays (Fig 1C; Table 1).^28^

**TABLE 1.**
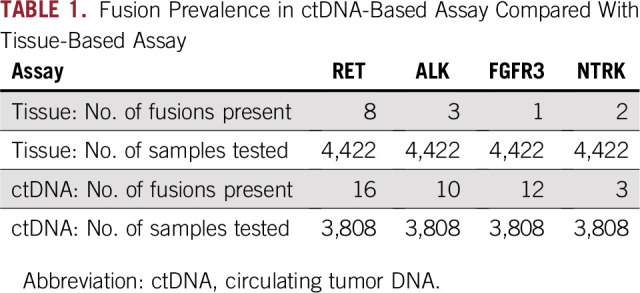
Fusion Prevalence in ctDNA-Based Assay Compared With Tissue-Based Assay

### Genomic Profiling of Fusion-Positive Patients

Clinicopathology history was available for a subset of patients (Table 2; Appendix Table A3). Because this was a retrospective review of clinically treated patients, tissue testing methodology varied over time and across different practices. At least some of the molecular data from tissue testing collected at the time of initial diagnosis was available for 24 of 40 ctDNA fusion-positive patients, eight of whom had comprehensive NGS in which the presence of fusions was assessed. The median time between tissue testing and ctDNA collection was 24.1 months (range, 0.67 to 92 months; n = 22). From the available clinical and tissue data, nine of 27 (33%) were right-sided, tumors were predominantly *KRAS* wild type (n = 23 of 24; 96%), with no concurrent *NRAS* or *BRAF^V600E^* mutations, and three of 22 (14%) were MSI-H (Table 2; Table A3). Interestingly, in 11 of the 23 patients with tissue *RAS* wild-type status, a *RAS* mutation was detected in ctDNA. Similarly, in two of the 16 patients with *BRAF*^V600E^ tissue wild-type status, *BRAF*^V600E^ was detected in ctDNA. Among the eight patients with tissue NGS available, only two had the matched fusion detected. Cumulatively, the data suggest that a sizable proportion of the ctDNA fusion-positive population may have had *RAS/RAF* mutations and/or the fusion present at levels below the limit of detection in tissue or in a subclone of the tumor tissue that was not sampled for testing.

**TABLE 2.**
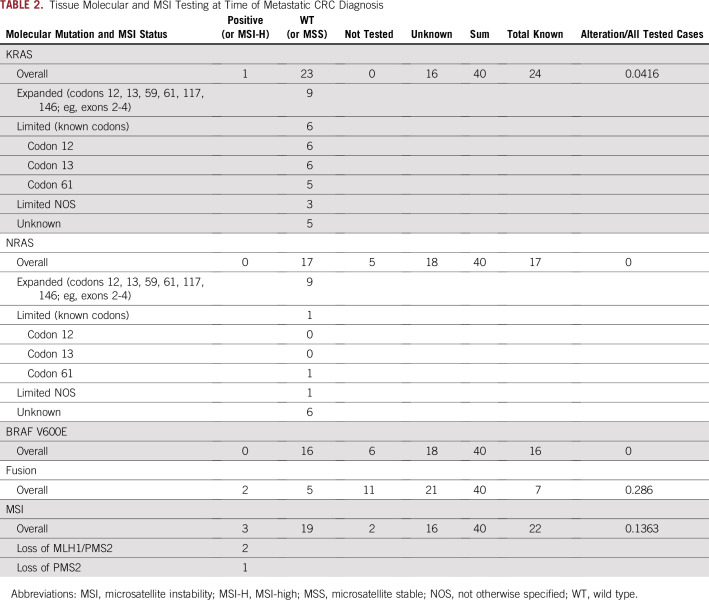
Tissue Molecular and MSI Testing at Time of Metastatic CRC Diagnosis

**FIG 2.**
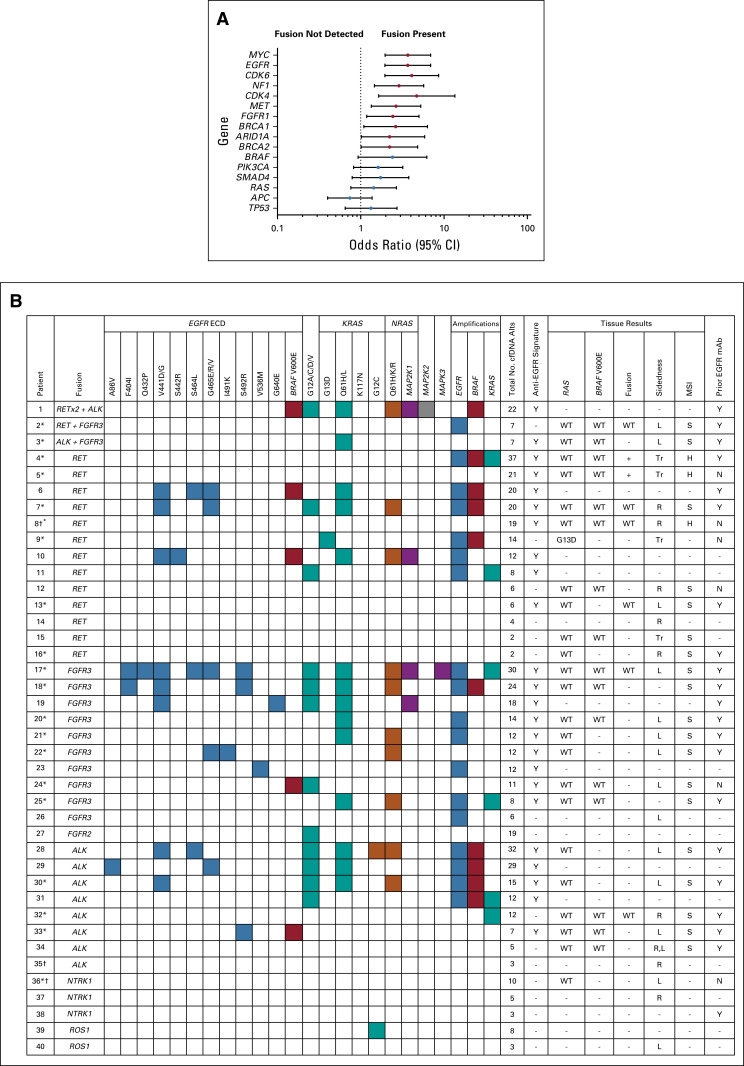
Co-occurring mutations in fusion patients. (A) Gene mutations associated with fusion presence. (B) *EGFR*, *RAS*, and *BRAF* amplifications, indels, and mutations occurring in fusion patients in circulating tumor DNA (ctDNA) and matched tissue samples. Blank cells indicate no mutation detected (for circulating free DNA [cfDNA] results) or not available (for tissue results and prior anti-epidermal growth factor receptor [EGFR] monoclonal antibody [mAb]). (*) Clinical history verified by ordering health care provider. (†) Patients had multiple samples drawn; figure provides a summary of unique alterations (alts) detected across all fusion-positive samples. +, KRAS G13D positive; ECD, extracellular domain; H, MSI-high; L, left; mAb, monoclonal antibody; MSI, microsatellite instability; N, no; R, right; Tr, transverse; S, MSI-stable; WT, wild type; Y, yes.

The frequency of amplifications, indels, and SNVs in clinically relevant cancer genes detectable using the blood-based NGS assay were compared between fusion-positive and fusion-negative samples (Fig 2A). There was no association between the presence of a fusion and coexisting mutation in *KRAS*, *NRAS*, or *BRAF*. Furthermore, co-occurring mutations were more likely in *EGFR* (odds ratio [OR], 3.66; 95% CI, 1.97 to 6.84; *P* < .001), *MET* (OR, 2.56; 95% CI, 1.30 to 5.04; *P <* .01), and *FGFR1* (OR, 2.46; 95% CI, 1.20 to 5.06; *P =* .01) for specimens with fusions, when compared with nonfusion cases (Fig 2A).

**FIG A1.**
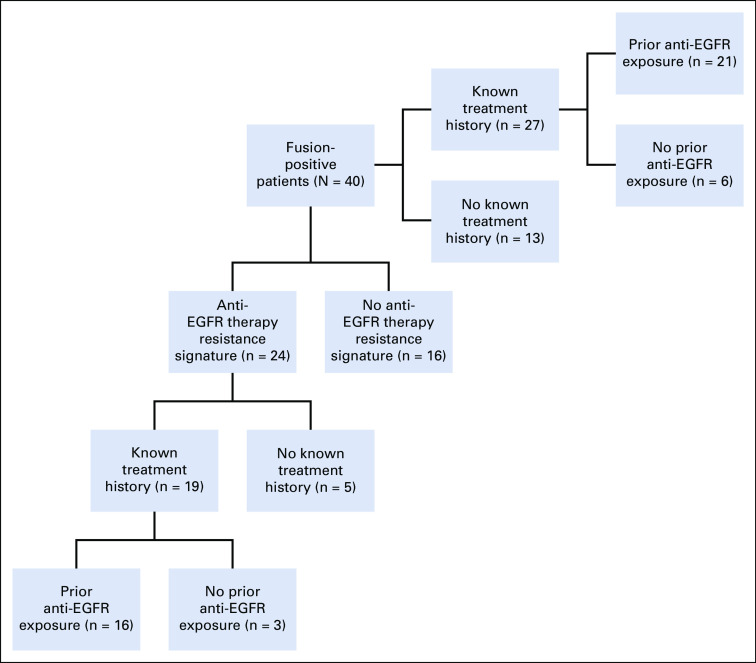
Consort diagram detailing fusion history and associated anti–epidermal growth factor receptor (anti-EGFR) therapy resistance signature.

Prior treatment histories were available for only 27 patients, the majority (n = 21; 78%) of whom did have prior exposure to one or more EGFR monoclonal antibodies as treatment of metastatic CRC at the time of ctDNA collection (Appendix Table A4; Appendix Fig A1). Therefore, we next explored if fusions were associated with a previously validated genomic signature associated with CRC progression on prior anti-EGFR therapies, because treatment histories were not available for the entire fusion cohort.^16,29^

### Anti-EGFR Signature

ctDNA genomic features of progression on prior cetuximab or panitumumab include the presence of subclonal *RAS* mutation (VAF < 50% of the maximum VAF in the sample), multiple concurrent *RAS* mutations, and/or *EGFR* mutations.^29^ In a previously validated large cohort of patients with metastatic CRC with and without anti-EGFR exposure, the presence of any one of these variables was highly predictive of prior anti-EGFR exposure (positive predictive value, 98.3%; specificity, 98.7%).^29^

In this series, 24 of 40 (60%) fusion-positive patients had subclonal *RAS* mutation (rVAF of < 50%), any *EGFR* mutation, or multiple concurrent *RAS* mutations. Fifteen of 40 (38%) had two or more of these. Mutations associated with this anti-EGFR therapy resistance signature were found with fusions in *FGFR3* (10 of 12 patients), *RET* (nine of 16 patients), and *ALK* (seven of 10 patients), including two of the patients with multiple fusions (Fig 2B). Among the 24 fusion-positive patients with mutations associated with this anti-EGFR therapy resistance signature, 19 had known treatment histories (Fig A1). Of these 19 patients, 16 (84%) patients were confirmed to have prior exposure to anti-EGFR therapy. The median duration of exposure to treatment with an anti-EGFR agent was 8.5 months (range, 2 to 17 months; Appendix Tables A4 and A5).

**FIG 3.**
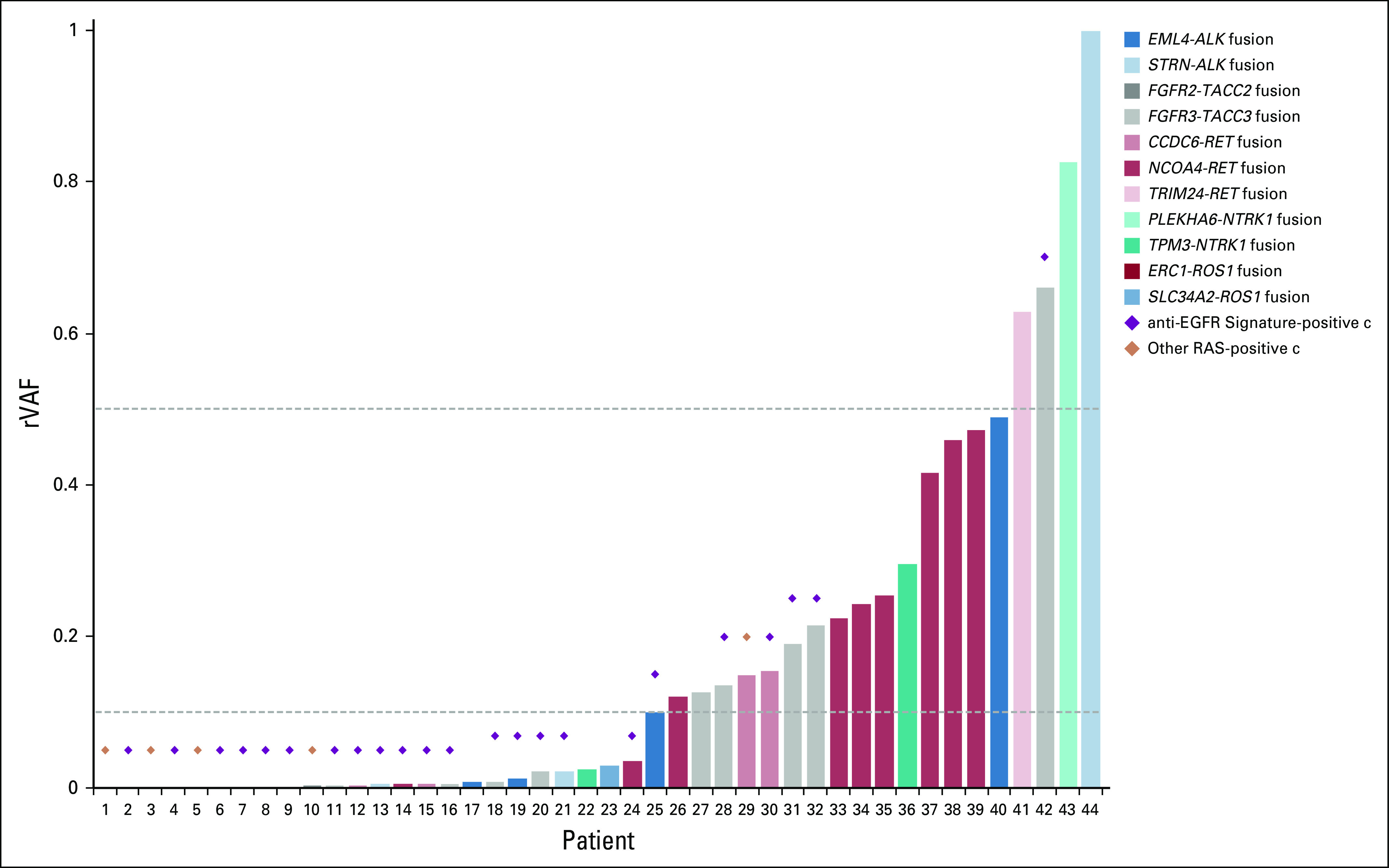
Distribution of copy-number adjusted relative variant allele fraction (rVAF) for fusion-present samples. Values above horizontal line at rVAF 0.5 indicate clonal fusions, values between 0.1 and 0.5 indicate subclonal fusions, and values below horizontal line at 0.1 indicate subclonal minor fusions.

**FIG A2.**
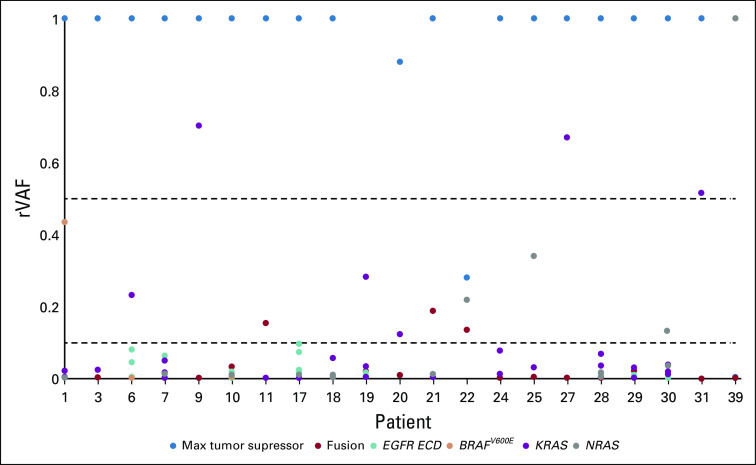
Relative variant allele fraction (rVAF) for 21 fusion-positive samples with *RAS* mutations and co-occurring *EGFR* extracellular domain (ECD) and *BRAF*^V600E^ mutations.

The presence of an anti-EGFR signature was associated with fusions occurring at lower rVAF (median, 0.01 *v* 0.19; *P* = .036; Fig 3). Furthermore, the low rVAFs of co-occurring *RAS*, *EGFR*, and *BRAF*^V600E^ mutations were consistent with subclonal genomic events occurring later in tumorigenesis (Appendix Fig A2). Among the six patients with an anti-EGFR resistance signature who had comprehensive genomic profiling results available from tissue, four were wild type at the time of initial diagnosis of CRC for the corresponding fusion and/or *RAS/RAF* alterations that were later detected in ctDNA, consistent with these genomic events being acquired later in tumorigenesis.

## DISCUSSION

To our knowledge, this is the largest case series describing fusion-positive cases in CRC (whether in tissue or plasma) and demonstrates that fusions in patients with CRC can be identified using a ctDNA assay. Here, fusions were detected at a prevalence of approximately 1% in patients with advanced CRC, similar to fusion prevalence using orthogonal tissue-based assays in separate series of patients with CRC.^8,9^ All fusions identified are potentially actionable with available targeted drugs. Thus, this ctDNA approach has the potential to allow clinicians to consider additional studies with novel therapeutic combinations for patients with metastatic CRC in future trial settings.

Our data provide new evidence that fusions, particularly involving *FGFR3* or *RET*, may contribute to anti-EGFR therapy resistance in CRC. Here, the majority of the fusions were subclonal. On the basis of previously validated genomic signatures in this setting, we hypothesize that fusions may arise as a novel, unreported mechanism with anti-EGFR therapy resistance, given the clinicopathologic data and frequent co-occurrence with subclonal *RAS* and *EGFR* mutations in ctDNA. The profile of concomitant *EGFR* mutations and subclonal *RAS* mutations mirrors prior studies that have shown associations between these mutations and post-EGFR resistance.^29^ Interestingly, prior series performed in tissue have associated fusions with *RAS* wild-type CRC tumors.^9-12^ In our series, 23 of 24 (96%) of the ctDNA fusion–positive patients with tissue testing available for *RAS* mutational status were *RAS* wild type, whereas 25 of 44 (57%) of fusion-positive ctDNA samples in our series had one or more *RAS* mutations. We reconcile these findings on the basis of the greater sensitivity to detect low allele frequency often not detectable with tissue-based assays. Furthermore, tissue specimens are often obtained at surgical resections, before a multiple number of sequential lines of systemic therapy, and therefore before exposure to selective pressures that mediate acquisition of resistance mechanisms. The majority of blood samples obtained in this cohort of patients with CRC came from treatment-refractory individuals seeking clinical trial options who frequently had been exposed to anti-EGFR therapies. Thus, the occurrence of subclonal resistance alterations in ctDNA accounted for differences in the tumor genomic profiles of advanced, typically heavily pretreated cancers, relative to the less-mutated genomic profiles of the tumor taken before therapy initiation. In this series, only one patient had a fusion detected in pretreatment tumor tissue and subsequently had anti-EGFR therapy but did not have a clinical response. Additional investigation into whether fusions also cause primary resistance to anti-EGFR therapy is warranted.

To lend additional support to this association between fusions and resistance to anti-EGFR antibodies, we confirmed the clinical histories of patients with fusion-detected CRC. In those patients with prior treatment data available, 21 of 27 (78%) had previous exposure and progression on anti-EGFR antibodies. Thus, these data further support the notion that subclonal fusions, here identified by ctDNA, may arise after treatment with anti-EGFR antibodies and may represent a novel mechanism of resistance in CRC to these agents.

Our findings in CRC are consistent with previously reported series linking activating fusions as mechanisms of acquired resistance to targeted therapies in other malignancies.^21-24^ For example, *RET* fusions were found in patients with NSCLC after the EGFR tyrosine kinase inhibitor (TKI) osimertinib.^23^ Previous studies have shown that *FGFR3* fusions may substitute for EGFR signaling, which provides a hypothesized rationale for a mechanism of acquired resistance to anti-EGFR therapy.^21^ All of the fusions detected in this series are predicted to lead to the generation of a chimeric protein involving fusion of a tyrosine kinase domain with a partner protein that enhances its activation, thereby promoting downstream signaling of the mitogen-activated protein kinase (MAPK) pathway.^30,31^ Activation of this alternative MAPK signaling pathway bypasses the reduction in MAPK signaling afforded by anti-EGFR antibodies, thus providing plausible biologic rationale for the association of fusion anti-EGFR therapy resistance.^32^

Furthermore, alterations in *MET* and *FGFR1* were also observed more commonly in patients with fusions. Such alterations have been previously reported as acquired mechanisms of resistance to anti-EGFR therapies in CRC.^20,33^ Collectively these and our data point to a diverse, heterogeneous landscape of potential resistance mechanisms adapted by *RAS* wild-type CRC tumors to overcome EGFR blockade.

Fusions represent a potentially actionable therapeutic target in the anti-EGFR resistance setting. Dual pathway suppression with the RET inhibitor BLU-667 and an EGFR TKI demonstrated antitumor activity both in cell lines and clinically in patients with EGFR-mutant NSCLC who had *RET* fusions after disease progression while receiving TKIs.^23^ Importantly, fusions were often seen co-occurring with multiple other known acquired mechanisms of resistance to anti-EGFR therapy in this series, which points to a diverse, heterogeneous landscape of potential resistance mechanisms adapted by *RAS* wild-type CRC tumors to overcome EGFR blockade. Therefore, although targeting subclonal fusions alone may be only partially successful, multipathway suppression may be a promising avenue of additional investigation, possibly in combination with anti-EGFR therapies. Such strategies would need to be highly individualized, given the diversity of resistance mechanisms, and could be informed by comprehensive ctDNA testing, especially because serial tissue biopsies are less feasible in patients with advanced cancer.

In several previous data sets using tissue-based assays, fusions in patients with CRC were associated with MSI-H cancers.^9-12^ Although rates of MSI-H and right-sided tumors in our data set were similar to average rates reported in advanced CRCs, a proportion of the fusion-positive patients in this series are suspected to have acquired the fusion after selective pressure from anti-EGFR therapy, and therefore the fusion may have been present in the primary tumor at levels too low to be associated with MSI-H status. In both cases in this series where the fusion was tested for and detected in tissue, the tumors were found to be MSI-H.

One of the limitations of this data analysis is that complete clinicopathologic features were not available for all patients, given the retrospective nature of the study, and therefore we were unable to obtain clinical histories from all patients with fusions. However, using a previously validated method,^29^ the majority of fusion-positive patients had at least one variable, which was highly predictive of prior anti-EGFR exposure. In addition, among the patients with known treatment history and this signature, the majority were indeed confirmed to have prior anti-EGFR therapy, thus internally validating the efficacy of this genomics-first strategy to identify likely resistance cases.

We also did not have matched pre- and post-treatment tissue and plasma for orthogonal and serial profiling to confirm which fusions and other co-occurring mutations were acquired/selected for after anti-EGFR therapy versus those present as truncal/clonal events. For the majority of our patients, we do not have access to the tissue or pretreatment plasma for additional NGS analysis based on the retrospective nature of study. However, genomic events that are acquired during cancer progression tend to have lower relative VAF in ctDNA than do early truncal mutations, such as those in tumor suppressor genes or clonal RAS mutations.^13^ In our series, fusions occurring at low rVAF tended to be found in samples containing other genomic mechanisms of anti-EGFR therapy resistance, which is consistent with our hypothesis that some fusions in CRC occur at subclonal levels that are undetectable in pretreatment tissue but are selected for and become detectable in ctDNA after anti-EGFR therapy resistance. The quantitative nature of ctDNA can therefore not only characterize the fusion identity but also provide insight into the clonal contribution via a single blood draw. Another limitation is that the VAF may be affected by biologic factors, such as the degree of tumor shedding, as well as technical factors, including that fusions are more difficult to detect by NGS and in ctDNA samples than SNVs. Taken together, the fusion prevalences and VAFs observed in this study may be lower than actual because of these technical reasons.

In conclusion, actionable fusions were able to be detected at low frequencies but at similar frequencies to the historical tissue-based NGS approach in a large series of patients with CRC using a ctDNA assay. The distribution of coexisting subclonal mutations in *EGFR*, *KRAS*, and *NRAS* in fusion-present CRC cases matches genomic profiles of CRC tumors after progression on prior anti-EGFR therapy in tumors initially identified as *RAS* wild type using a less-sensitive tissue-based assay. Actionable fusions may therefore represent a newly reported mechanism of acquired resistance after anti-EGFR therapies. Testing ctDNA in patients to detect fusions as targetable drivers and/or resistance biomarkers is warranted and may carry important implications for the treating oncologist to identify novel therapeutic approaches.

## Figures and Tables

**TABLE A1.**
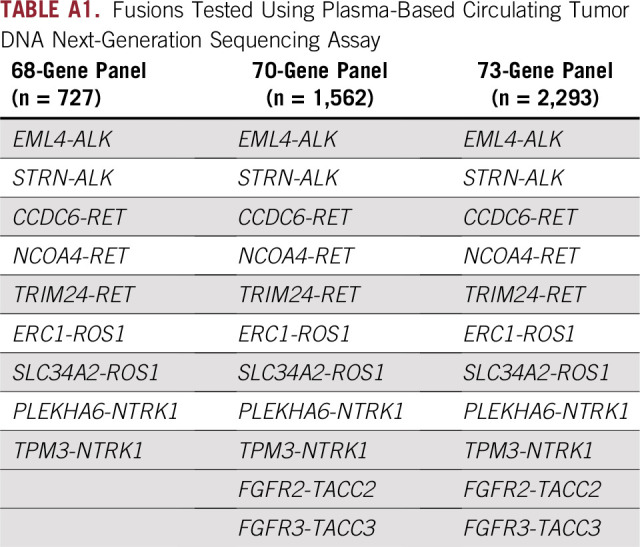
Fusions Tested Using Plasma-Based Circulating Tumor DNA Next-Generation Sequencing Assay

**TABLE A2.**
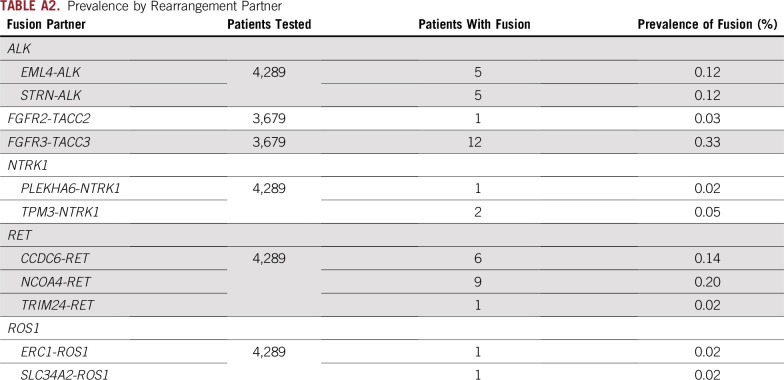
Prevalence by Rearrangement Partner

**TABLE A3.**
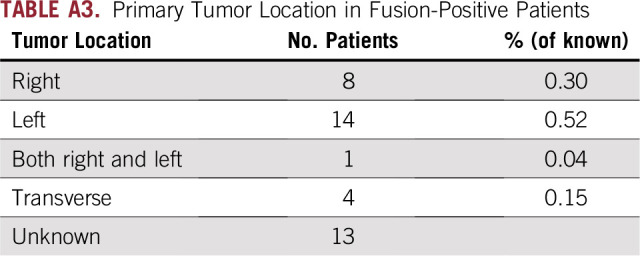
Primary Tumor Location in Fusion-Positive Patients

**TABLE A4.**
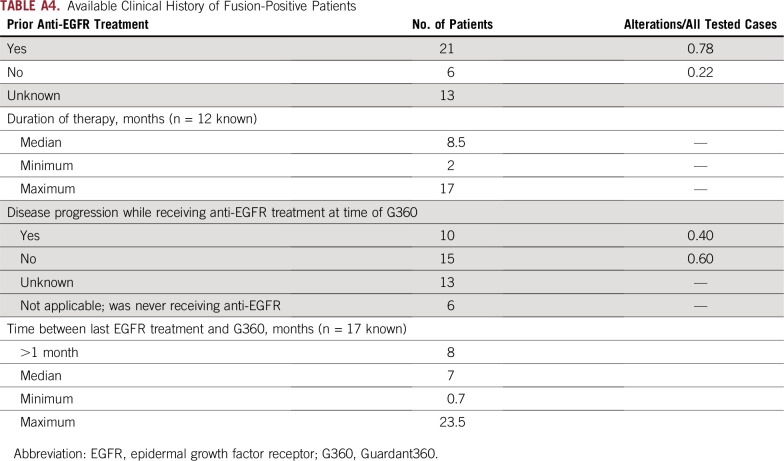
Available Clinical History of Fusion-Positive Patients

**TABLE A5.**
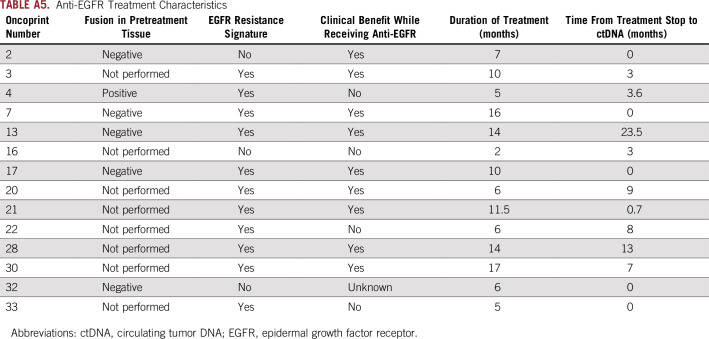
Anti-EGFR Treatment Characteristics

## References

[1] Medves S Demoulin JB Tyrosine kinase gene fusions in cancer: Translating mechanisms into targeted therapies J Cell Mol Med 16 237 248 2012 21854543 10.1111/j.1582-4934.2011.01415.x PMC3823288

[2] Schram AM Chang MT Jonsson P et al Fusions in solid tumours: Diagnostic strategies, targeted therapy, and acquired resistance Nat Rev Clin Oncol 14 735 748 2017 28857077 10.1038/nrclinonc.2017.127 PMC10452928

[3] Drilon A Laetsch TW Kummar S et al Efficacy of larotrectinib in TRK fusion-positive cancers in adults and children N Engl J Med 378 731 739 2018 29466156 10.1056/NEJMoa1714448 PMC5857389

[4] Yakirevich E Resnick MB Mangray S et al Oncogenic ALK fusion in rare and aggressive subtype of colorectal adenocarcinoma as a potential therapeutic target Clin Cancer Res 22 3831 3840 2016 26933125 10.1158/1078-0432.CCR-15-3000

[5] Amatu A Somaschini A Cerea G et al Novel CAD-ALK gene rearrangement is drugable by entrectinib in colorectal cancer Br J Cancer 113 1730 1734 2015 26633560 10.1038/bjc.2015.401 PMC4701996

[6] Subbiah V Gainor JF Rahal R et al Precision targeted therapy with BLU-667 for *RET*-driven cancers Cancer Discov 8 836 849 2018 29657135 10.1158/2159-8290.CD-18-0338

[7] Subbiah V Velcheti V Tuch BB et al Selective RET kinase inhibition for patients with RET-altered cancers Ann Oncol 29 1869 1876 2018 29912274 10.1093/annonc/mdy137 PMC6096733

[8] Stransky N Cerami E Schalm S et al The landscape of kinase fusions in cancer Nat Commun 5 4846 2014 25204415 10.1038/ncomms5846 PMC4175590

[9] Cocco E Benhamida J Middha S et al Colorectal carcinomas containing hypermethylated MLH1 promoter and wild type BRAF/KRAS are enriched for targetable kinase fusions Cancer Res 79 1047 1053 2019 30643016 10.1158/0008-5472.CAN-18-3126 PMC6420871

[10] Pietrantonio F, Di Nicolantonio F, Schrock AB, et al: ALK, ROS1, and NTRK rearrangements in metastatic colorectal cancer. J Natl Cancer Inst 10.1093/jnci/djx089 29370427

[11] Pietrantonio F Di Nicolantonio F Schrock AB et al RET fusions in a small subset of advanced colorectal cancers at risk of being neglected Ann Oncol 29 1394 1401 2018 29538669 10.1093/annonc/mdy090

[12] Wang J Yi Y Xiao Y et al Prevalence of recurrent oncogenic fusion in mismatch repair-deficient colorectal carcinoma with hypermethylated MLH1 and wild-type BRAF and KRAS Mod Pathol 32 1053 1064 2019 30723297 10.1038/s41379-019-0212-1

[13] Zill OA Banks KC Fairclough SR et al The landscape of actionable genomic alterations in cell-free circulating tumor DNA from 21,807 advanced cancer patients Clin Cancer Res 24 3528 3538 2018 29776953 10.1158/1078-0432.CCR-17-3837

[14] Diaz LA Jr Sausen M Fisher GA et al Insights into therapeutic resistance from whole-genome analyses of circulating tumor DNA Oncotarget 4 1856 1857 2013 24196513 10.18632/oncotarget.1486 PMC3858570

[15] Misale S, Yaeger R, Hobor S et al: Emergence of KRAS mutations and acquired resistance to anti-EGFR therapy in colorectal cancer. Nature 486:532-536, 2012 10.1038/nature11156 PMC3927413 22722830

[16] Strickler JH Loree JM Ahronian LG et al Genomic landscape of cell-free DNA in patients with colorectal cancer Cancer Discov 8 164 173 2018 29196463 10.1158/2159-8290.CD-17-1009 PMC5809260

[17] Diaz LA Jr Williams RT Wu J et al The molecular evolution of acquired resistance to targeted EGFR blockade in colorectal cancers Nature 486 537 540 2012 22722843 10.1038/nature11219 PMC3436069

[18] Bardelli A Corso S Bertotti A et al Amplification of the MET receptor drives resistance to anti-EGFR therapies in colorectal cancer Cancer Discov 3 658 673 2013 23729478 10.1158/2159-8290.CD-12-0558 PMC4078408

[19] Siravegna G, Mussolin B, Buscarino M, et al: Clonal evolution and resistance to EGFR blockade in the blood of colorectal cancer patients. Nat Med 21:795-801, 2015 [Erratum: Nat Med 21:827, 2015] 10.1038/nm.3870 PMC4868598 26030179

[20] Bertotti A Papp E Jones S et al The genomic landscape of response to EGFR blockade in colorectal cancer Nature 526 263 267 2015 26416732 10.1038/nature14969 PMC4878148

[21] Daly C Castanaro C Zhang W et al FGFR3-TACC3 fusion proteins act as naturally occurring drivers of tumor resistance by functionally substituting for EGFR/ERK signaling Oncogene 36 471 481 2017 27345413 10.1038/onc.2016.216 PMC5290037

[22] McCoach CE Blakely CM Banks KC et al Clinical utility of cell-free DNA for the detection of *ALK* fusions and genomic mechanisms of ALK inhibitor resistance in non-small cell lung cancer Clin Cancer Res 24 2758 2770 2018 29599410 10.1158/1078-0432.CCR-17-2588 PMC6157019

[23] Piotrowska Z Isozaki H Lennerz JK et al Landscape of acquired resistance to osimertinib in EGFR-mutant NSCLC and clinical validation of combined EGFR and RET inhibition with osimertinib and BLU-667 for acquired RET fusion Cancer Discov 8 1529 1539 2018 30257958 10.1158/2159-8290.CD-18-1022 PMC6279502

[24] Liang W He Q Chen Y et al Metastatic EML4-ALK fusion detected by circulating DNA genotyping in an EGFR-mutated NSCLC patient and successful management by adding ALK inhibitors: A case report BMC Cancer 16 62 2016 26850068 10.1186/s12885-016-2088-5 PMC4744376

[25] Lanman RB Mortimer SA Zill OA et al Analytical and clinical validation of a digital sequencing panel for quantitative, highly accurate evaluation of cell-free circulating tumor DNA PLoS One 10 e0140712 2015 26474073 10.1371/journal.pone.0140712 PMC4608804

[26] Odegaard JI Vincent JJ Mortimer S et al Validation of a plasma-based comprehensive cancer genotyping assay utilizing orthogonal tissue- and plasma-based methodologies Clin Cancer Res 24 3539 3549 2018 29691297 10.1158/1078-0432.CCR-17-3831

[27] Hu Y Ulrich BC Supplee J et al False-positive plasma genotyping due to clonal hematopoiesis Clin Cancer Res 24 4437 4443 2018 29567812 10.1158/1078-0432.CCR-18-0143

[28] Rankin A Klempner SJ Erlich R et al Broad detection of alterations predicted to confer lack of benefit from EGFR antibodies or sensitivity to targeted therapy in advanced colorectal cancer Oncologist 21 1306 1314 2016 27682134 10.1634/theoncologist.2016-0148 PMC5189622

[29] Parseghian CM Loree JM Morris VK et al Anti-EGFR-resistant clones decay exponentially after progression: Implications for anti-EGFR re-challenge Ann Oncol 30 243 249 2019 30462160 10.1093/annonc/mdy509 PMC6657008

[30] Chae YK Ranganath K Hammerman PS et al Inhibition of the fibroblast growth factor receptor (FGFR) pathway: The current landscape and barriers to clinical application Oncotarget 8 16052 16074 2017 28030802 10.18632/oncotarget.14109 PMC5362545

[31] Bunone G Uggeri M Mondellini P et al RET receptor expression in thyroid follicular epithelial cell-derived tumors Cancer Res 60 2845 2849 2000 10850426

[32] Oda K Matsuoka Y Funahashi A Kitano H A comprehensive pathway map of epidermal growth factor receptor signaling Mol Syst Biol 1 1 17 2005 10.1038/msb4100014 PMC1681468 16729045

[33] Raghav K Morris V Tang C et al MET amplification in metastatic colorectal cancer: An acquired response to EGFR inhibition, not a de novo phenomenon Oncotarget 7 54627 54631 2016 27421137 10.18632/oncotarget.10559 PMC5342368

